# Speed and efficiency: evaluating pulmonary nodule detection with AI-enhanced 3D gradient echo imaging

**DOI:** 10.1007/s00330-024-11027-5

**Published:** 2024-08-18

**Authors:** Sebastian Ziegelmayer, Alexander W. Marka, Maximilian Strenzke, Tristan Lemke, Hannah Rosenkranz, Bernadette Scherer, Thomas Huber, Kilian Weiss, Marcus R. Makowski, Dimitrios C. Karampinos, Markus Graf, Joshua Gawlitza

**Affiliations:** 1 Department of Diagnostic and Interventional Radiology, School of Medicine & Klinikum rechts der Isar, Technical University of Munich, Munich, Germany; 2 Philips GmbH Market DACH, Hamburg, Germany

**Keywords:** Lung, Multiple pulmonary nodules, Magnetic resonance images, Computed tomography

## Abstract

**Objectives:**

Evaluating the diagnostic feasibility of accelerated pulmonary MR imaging for detection and characterisation of pulmonary nodules with artificial intelligence-aided compressed sensing.

**Materials and methods:**

In this prospective trial, patients with benign and malignant lung nodules admitted between December 2021 and December 2022 underwent chest CT and pulmonary MRI. Pulmonary MRI used a respiratory-gated 3D gradient echo sequence, accelerated with a combination of parallel imaging, compressed sensing, and deep learning image reconstruction with three different acceleration factors (CS-AI-7, CS-AI-10, and CS-AI-15). Two readers evaluated image quality (5-point Likert scale), nodule detection and characterisation (size and morphology) of all sequences compared to CT in a blinded setting. Reader agreement was determined using the intraclass correlation coefficient (ICC).

**Results:**

Thirty-seven patients with 64 pulmonary nodules (solid *n* = 57 [3–107 mm] part-solid *n* = 6 [ground glass/solid 8 mm/4–28 mm/16 mm] ground glass nodule *n* = 1 [20 mm]) were analysed. Nominal scan times were CS-AI-7 3:53 min; CS-AI-10 2:34 min; CS-AI-15 1:50 min. CS-AI-7 showed higher image quality, while quality remained diagnostic even for CS-AI-15. Detection rates of pulmonary nodules were 100%, 98.4%, and 96.8% for CS-AI factors 7, 10, and 15, respectively. Nodule morphology was best at the lowest acceleration and was inferior to CT in only 5% of cases, compared to 10% for CS-AI-10 and 23% for CS-AI-15. The nodule size was comparable for all sequences and deviated on average < 1 mm from the CT size.

**Conclusion:**

The combination of compressed sensing and AI enables a substantial reduction in the scan time of lung MRI while maintaining a high detection rate of pulmonary nodules.

**Clinical relevance statement:**

Incorporating compressed sensing and AI in pulmonary MRI achieves significant time savings without compromising nodule detection or characteristics. This advancement holds clinical promise, enhancing efficiency in lung cancer screening without sacrificing diagnostic quality.

**Key Points:**

- *Lung cancer screening by MRI may be possible but would benefit from scan time optimisation*.
- *Significant scan time reduction, high detection rates, and preserved nodule characteristics were achieved across different acceleration factors*.
- *Integrating compressed sensing and AI in pulmonary MRI offers efficient lung cancer screening without compromising diagnostic quality.*

## Introduction

The substantial decrease in lung cancer-related deaths achieved through low-dose computed tomography (CT) screening has been proven by several studies and led to the widespread adoption of nationwide screening programs that have already been implemented or are on the verge of happening in numerous countries [1–3]. Although the cumulative radiation dose associated with low-dose CT of the thorax in annual screening is acceptable, high false positive rates [3–5] can result in a substantial increase in radiation dose individually [6] through further diagnostic workup and patients may be mistakenly subjected to invasive workup. Furthermore, the potentially harmful radiation associated with CT is one of the factors why patients do not want to participate in screening [7, 8]. Interestingly, Allen et al showed that magnetic resonance imaging (MRI) is potentially a cost-effective alternative to low-dose CT for lung cancer screening, using data from an intervention trial that investigated MRI as a screening method [9].

After overcoming the technical limitations, for example, strong movement and pulsation artefacts in the thorax, MRI might be well suited as a radiation-free alternative for lung cancer screening, as postulated in a position paper of the Fleischner Society [10]. However, MRI screening would further increase the already high infrastructural requirements of lung cancer screening [11]. Besides the technical and monetary challenges, the scan duration is another particular hurdle compared to CT in a nationwide screening program. Shorter examination times with sufficient image quality would be crucial to allow a high patient throughput. Compressed sensing and artificial intelligence (CS-AI) acceleration have been used in combination in a variety of MR imaging areas. Especially in cardiac or musculoskeletal imaging [12, 13], it has shown great potential by reducing data acquisition time and improving image reconstruction.

Therefore, the aim of this study was to evaluate a 3D gradient echo sequence with three different acceleration factors and compare image quality and diagnostic performance in nodule detection and characterisation with the gold-standard CT.

## Material and methods

### Patients

The study was designed as a single-centre prospective study at our institution. It was approved by the local ethical review board (protocol number 692/21S). All participants gave written informed consent. Patients undergoing chest CT in clinical routine between December 2021 and December 2022 were examined for eligibility. Patients’ inclusion criteria were (1) chest CT, (2) evidence of solid, part-solid or ground glass lung nodules > 3 mm, regardless of aetiology, and (3) interval between the first CT and available MRI scan slot less than seven days. Individuals with conditions excluded by MR safety guidelines such as pacemakers, other implanted electronic devices or pregnancy, were not included. Additionally, patients with lung parenchyma pathology such as infiltration, oedema, pleural effusion, or emphysema were excluded from the study. Lastly, patients in which the aetiology could not be clarified during the study interval were excluded.

### CT image acquisition

All patients underwent thin-section chest CT with a mean tube current of 59 mA for standard CT on a 320-detector row CT scanner (IQon—Spectral CT, Philips Healthcare). Tube voltage was 120 kV, mean tube current (59 mAs), collimation 64 × 0.625, pitch factor 1. Images were reconstructed using a dedicated lung kernel (YA) with an iterative reconstruction level of 6 and a slice thickness of 1 mm. Scans were performed at inspiration.

### MR image acquisition

All MR images were acquired on a 3-T MR scanner (Ingenia Elition X; Philips Healthcare) using a combination of a 16-element phased-array anterior and a built-in 12-element phased-array posterior coil. The MR images were acquired using three different respiratory-gated, accelerated 3D gradient echo sequences with isotropic resolution. Data acquisition was based on a spoiled gradient echo sequence using non-selective RF excitation combined with partial Fourier frequency encoding to minimize echo and repetition times. Image acceleration was based on a cartesian balanced variable density incoherent *k*-space sampling pattern with high sampling density in the *k*-space centre and continuously decreasing sampling density towards the *k*-space periphery in combination with a reconstruction algorithm, combining parallel imaging, CS-AI. The examination protocol included the three CS-AI sequences with different acceleration factors: (1) with an acceleration factor of 7 reconstructed using CS-AI (CS-AI-7) (repetition time: 2.7 ms; echo time: 0.83 ms; flip angle: 10°; FOV: 320 × 557 × 320 mm^3^; acquired voxel size: 1 × 1 × 1 mm^3^; reconstructed voxel size: 0.5 × 0.5 × 0.5 mm^3^; acceleration factor 7; nominal scan time 233 s), (2) with an acceleration factor of 10 reconstructed using CS-AI (CS-AI-10) (repetition time: 2.7 ms; echo time: 0.83 ms; flip angle: 10°; FOV: 320 × 557 × 320 mm^3^; acquired voxel size: 1 × 1 × 1 mm^3^; reconstructed voxel size: 0.5 × 0.5 × 0.5 mm^3^; acceleration factor 10; nominal scan time 164), and (3) with an acceleration factor of 15 reconstructed using CS-AI (CS-AI-15) (repetition time: 2.7 ms; echo time: 0.83 ms; flip angle: 10°; FOV: 320 × 557 × 320 mm^3^; acquired voxel size: 1 × 1 × 1 mm^3^; reconstructed voxel size: 0.5 × 0.5 × 0.5 mm^3^; acceleration factor 15; nominal scan time 90 s).

### MR image reconstruction

Image reconstruction was based on the Adaptive-CS-Net architecture as presented by Pezzotti et al [14]. The Adaptive-CS-Network mimics the iterative shrinkage–thresholding algorithm approach presented by Zhang et al [15] and integrates multiscale sparsification in a problem-specific learnable manner and combines a convolutional neural network-based sparsifying transformation with the image reconstruction approach of compressed sense [16–18], which ensures data consistency and incorporates domain-specific prior knowledge such as coil sensitivity distribution and location of the image background. Adaptive-CS-Net combines convolutional neural network-based deep learning with compressed sensing (CS) and parallel imaging for image reconstruction and was part of the international fast MRI challenge 2019 carried out by Facebook AI and New York University, where it scored best in the multi-coil categories with 4-fold and 8-fold undersampling [19]. The Adaptive-CS-Net was further developed and adapted by the manufacturer to include training data of more than 740,000 MR images of several contrast and anatomies from both 1.5 T and 3.0 T and to allow execution on standard reconstruction hardware and is referred to as SmartSpeed by the manufacturer (Philips Healthcare).

### Imaging analysis

CS-AI-7, CS-AI-10, and CS-AI-15 datasets, and CT images were analysed in a randomised order with an interval of 4 weeks between readings to prevent recall bias. MR images were analysed separately by two radiologists (9 years and 10 years of experience), blinded to all clinical and other information. Image quality was graded using an ordinal 5-point Likert scale (1 = non-diagnostic image quality with strong artefacts, 2 = severe blurring causing uncertain evaluation, 3 = moderate blurring that marginally restricts assessment, 4 = slight blurring of the structures that does not restrict image assessment, 5 = excellent image quality without any artefact) evaluating the following criteria: partial volume effect, blurring and discrimination from adjacent structures. The image quality was evaluated in sections from central to peripheral based on the vascular tree (main pulmonary artery; segmental vessels; subsegmental vessels; peripheral 1/3; subpleural and pleural vessels). Image quality scores of ≥ 4 were determined to be comparable to CT and a score ≥ 3 was defined as diagnostic. Further, the maximum diameter of each detected nodule was measured. Finally, the Lung-RADS classification was assessed for the CT and each MR sequence. Finally, the Lung-RADS (v. 2022) classification [20] was assessed for the CT and each MR sequence. After the last reading for each case, raters were given the three variations of the 3D gradient echo sequence with different acceleration factors, as well as the associated CT, to evaluate nodule morphology using an ordinal 3-point Likert scale (1 = equal, 2 = slightly inferior, and 3 = substantially inferior).

### Statistical analysis

Statistical analysis was performed using R and R-Studio. Patient and nodule characteristics are described in Table 1. Nodule detection rates were calculated for each sequence with the CT serving as the ground truth. To compare image quality each section was evaluated using the Kruskal–Wallis test. Significance tests were followed up by pairwise Wilcoxon tests. Furthermore, Bland–Altman diagrams were plotted to assess the mean differences and the 95% confidence intervals for the nodule maximum diameter. The agreement for the Lung-RADS categories was calculated for both readers between the CT and the respective MR sequences using weighted (squared) Cohen’s kappa. The nodule morphology was evaluated descriptively using three size thresholds (< 10 mm, 10–30 mm, > 30 mm). Lastly, the intraclass correlation coefficient (ICC) was calculated between the readers for each MR-Sequence and the CT. 95% confidence intervals were calculated by bias-corrected bootstrapping using 1000 replications.

**Table 1:** Patient characteristics

Characteristic	Value
Sex	
No. of men	21/37 (57%)
No. of women	16/37 (43%)
Age (y)*	66 ± 11 (40–84)
Time between CT and MRI (days)	4 ± 2 (1–7)
Lesion aetiology	*N* = 64
Metastasis	42
Primary lung cancer	8
Inflammatory	9
Granuloma	5
Nodule type	
Solid	57
Part-solid	6
Ground glass	1

## Results

### Participant and nodule characteristics

**Fig. 1:**
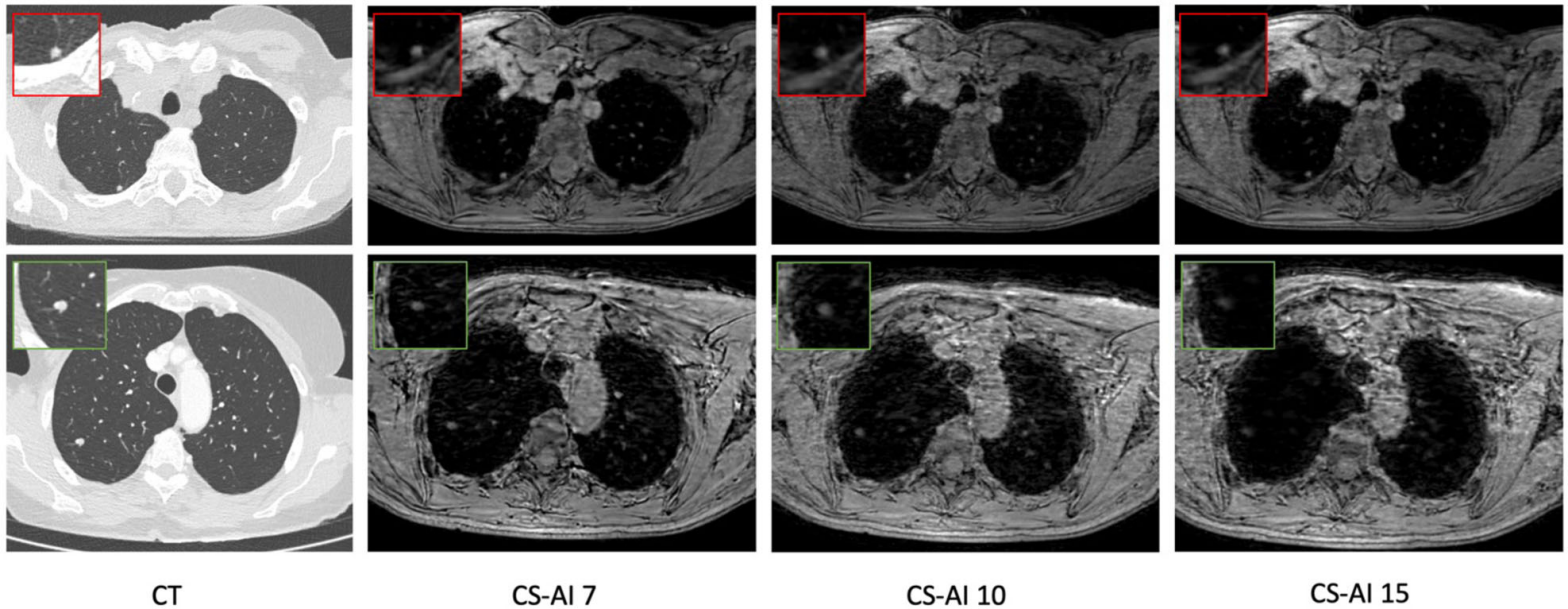
Exemplary CT images and MR images with different acceleration factors CS-AI-7, CS-AI-10, and CS-AI-15. The top row shows a case with a high score for image quality and morphology for all acceleration factors, whereas the bottom row shows a case with low image and morphology quality for CS-AI-10 (image quality 3; morphology slightly inferior) and 15 (image quality 2.5; morphology substantially inferior)

In this prospective study, 37 participants (mean age, 66 years (range 40–84 years); 16 women) with 64 nodules were evaluated. The aetiology of the lung nodules was metastases in 65.5%, 12.5% were primary lung cancer, and 22.0% were benign (inflammatory or granuloma). The aetiology of all primary lung cancers was histologically proven. All granulomas were reliably identified by follow-up and previous examinations. Metastases were reliably identified by follow-up and previous examinations in 32 cases and histologically proven in 10 cases. Nodule distribution was *n* = 57 solid, *n* = 6 part-solid, and *n* = 1 ground glass nodule. Nodule size was measured as the maximum diameter and ranged from 3 mm to 107 mm for solid nodules with a mean of 17 mm and a median of 12 mm. Part solid nodules had a size range depending on the ground-glass/solid component of 8 mm/4 mm to 28 mm/16 mm. The ground glass nodule had a maximum diameter of 20 mm. The pulmonary MRI with accelerated 3D T1 using CS and AI noise reduction was obtained within days after the initial low-dose CT (mean 4 days, ± 2 (1–7)). Details of patient characteristics are shown in Table 1. Two exemplary cases are illustrated in Fig. 1.

### Imaging analysis

**Fig. 2:**
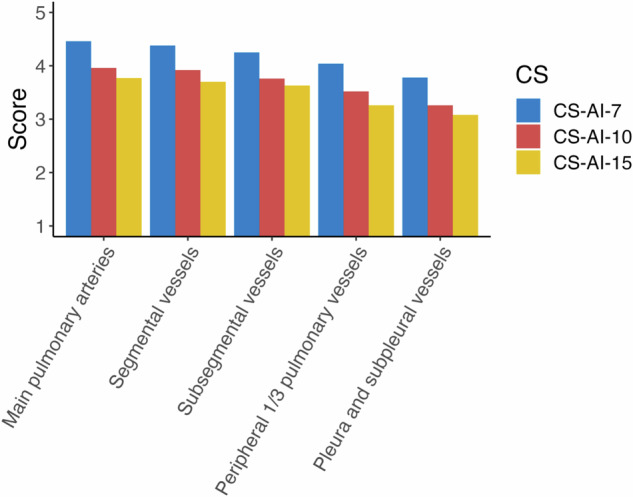
Grouped bar plots visualising the difference in image quality for anatomical landmarks. CS-AI-7 showed significantly higher image quality compared to CS-AI-10 and CS-AI-15 for all landmarks

Image quality was assessed individually for each section, a score ≥ 4 was comparable to CT and a score ≥ 3 was defined as diagnostic. The Kruskal–Wallis test showed that the lowest acceleration (CS-AI-7) achieved a significantly higher image quality for all sections (main pulmonary arteries *p* < 0.001; segmental vessels *p* < 0.001; subsegmental vessels *p* < 0.001; peripheral 1/3 pulmonary vessels *p* < 0.001; pleura and subpleural vessels *p* < 0.001). The results of the post hoc test can be found in supplementary material S1. An image quality comparable to CT was achieved for CS-AI-7 in 81%, for CS-AI-10 in 52% and for CS-AI-15 in 44% of the cases (Fig. 2). However, diagnostic quality was achieved for each anatomical structure in all sequences. The lowest acceleration achieved a detection rate of 100%, ensuring that no pulmonary nodule was missed. For the CS-AI-10 and -15 accelerations, the detection rate was 98.4% and 96.8%, respectively. As a result, one nodule was missed with the CS-AI-10 sequence and two nodules were missed with the CS-AI-15 sequence. The missed nodules were identical for both readers and were from the same case. The size of the missed nodule with both the CS-AI-10 and -15 sequences was 3 mm, and an additional 4 mm nodule was missed with the CS-AI-15. The image quality for the specific case was 3 for CS-AI-10 and CS-AI-15.

**Fig. 3:**
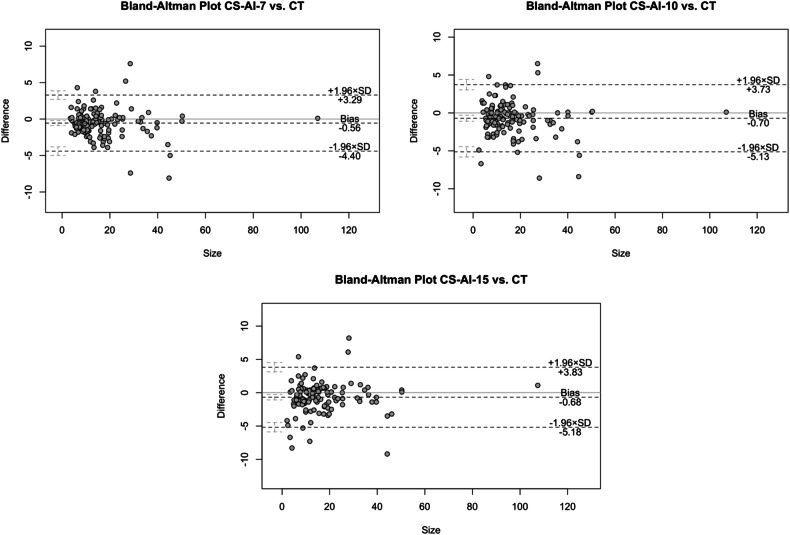
Bland–Altman diagrams showing the mean difference and the ± 1.96 standard deviation in maximum nodule diameter in millimetres between the respective acceleration factors and CT

Maximum nodule diameter was measured in all acceleration factors and the CT. The ICC was examined for both readers. The ICC showed excellent inter-rater agreement for the blinded reading (ICC-CT: 0.983, CI95: 0.953, 0.994; ICC-CS-AI-7: 0.985, CI95: 0.965, 0.993; ICC-CS-AI-10: 0.979, CI95: 0.949, 0.992; ICC-CS-AI-15: 0.982, and CI95: 0.951, 0.994). The mean maximum nodule diameter for CS-AI-7 deviated from CT of − 0.56 mm (1.96 SD 3.29 mm; − 1.96 SD − 4.40 mm). For CS-AI-10 and -15, the deviation was only slightly higher at − 0.70 mm (1.96 SD 3.73 mm; − 1.96 SD − 5.13 mm) and − 0.68 mm (1.96 SD 3.83 mm; − 1.96 SD 5.18 mm) and therefore less than 1 mm on average (Fig. 3).

For both readers Lung-RADS category agreement was almost perfect for all acceleration factors (CS-AI-7 = 0.96; CS-AI-10 = 0.95; CS-AI-15 = 0.94; all *p* < 0.001). Depending on the acceleration factor, there was a change in the Lung-RADS recommendation in three cases for CS-AI-7 (two upgraded 2 ≥ 3; 3 ≥ 4a and one downgraded 4X ≥ 4a), in six cases for CS-AI-10 (three upgraded 2 ≥ 3; − 3 > 4a; 4a ≥ 4b and two downgraded 4a ≥ 3; 4X ≥ 4a) and in four cases for CS-AI-15 (one upgraded 4a ≥ 4X and three downgraded 4a ≥ 3; 4X ≥ 4a; 4X ≥ 4a). The individual Lung-RADS categories for the MR sequences and the CT can be found in Table 2.

**Table 2:** Lung-RADS classification for CT and MR

Lung-RADS	CT	CS-AI 7	CS-AI 10	CS-AI 15
1	1	1	0	0
2	17	16	17	18
3	3	3	4	4
4A	3	5	3	3
4B	7	7	8	7
4X	7	6	6	6

**Fig. 4:**
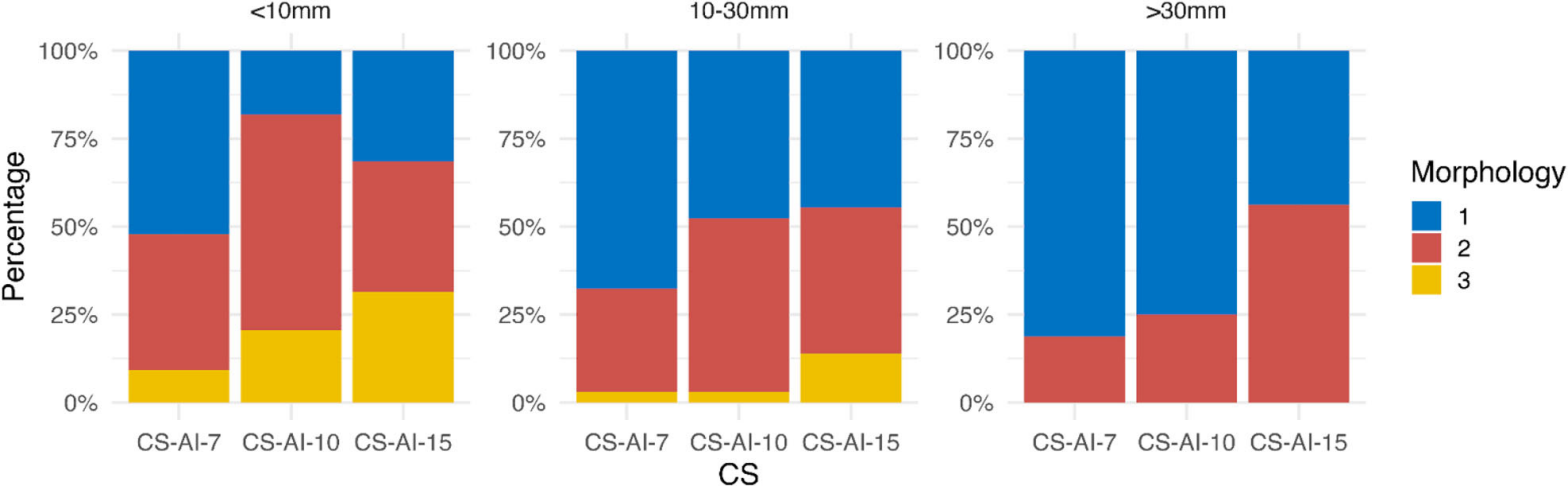
Percentage distribution of node morphology by acceleration factor (1 = identical to CT, 2 = slightly inferior, and 3 = substantially inferior)

Nodule morphology was assessed according to a 3-point Likert scale for three size thresholds (< 10 mm, 10–30 mm, > 30 mm). Similar to image quality, the lowest acceleration factor showed the best quality on average and was comparable to CT at 64% and only slightly inferior at 31%. For the CS-AI-10 and 15, the percentage of morphologically comparable nodules decreased to 40% and 25%, respectively, and slightly inferior was reported in 50% and 52% of the cases, respectively. Substantially worse evaluation of morphology was only 5% for CS-AI-7, 10% for CS-AI-10 and 23% for CS-AI-15. Using the size bins, a higher comparability of the sequences with the CT could be shown with increasing nodule size. Thus, the morphological comparability or slight inferiority with CS-AI-7 increased from 90.8% for nodules < 10 mm to 100% for nodules > 30 mm. A more pronounced increase was seen for CS-AI-10 and − 15 from 79.5% and 68.5%, respectively for nodules < 10 mm to 100% for nodules > 30 mm. The evaluation of morphology by size is shown in Fig. 4.

## Discussion

In our work, we were able to prove the diagnostic robustness of lung MRI for pulmonary nodule detection utilising a 3D gradient echo sequence with different acceleration factors using CS and deep learning image reconstruction. Although image quality and morphologic quality of the nodules decreased (Likert scale ≥ 3) with increasing scan acceleration, all evaluated structures remained of diagnostic quality, even for the highest acceleration factor (CS-AI-15). Further, a consistently high detection rate was achieved despite increasing acceleration (CS-AI-7 100%; CS-AI-10 98.4%; CS-AI 15–96.8%) and diametric deviation from CT as the gold standard was less than 1 mm for all acceleration factors.

In recent years, CS has reduced scan times in many areas without compromising image quality. In lung imaging, few studies have investigated methods to reduce scan time [21], in part because there is no established sequence standard. To our knowledge, our work is the first to investigate the effect of different acceleration factors on gradient echo sequences to detect pulmonary nodules. It was found that the image quality and quality of nodule morphology was lower with increasing acceleration compared to CT, but remained diagnostic for all evacuated structures even at the highest acceleration. This is in contrast to previous studies, which showed no deterioration in image quality even at higher acceleration factors [22], which can be attributed to the comparison with only compressed sense accelerated images in the aforementioned studies. However, the aim of our work was to achieve a balance between maximum reduction in scan time and sufficient image quality with regard to a potential application in lung cancer screening.

For nodule detection promising results have been obtained for using T2-weighted half-Fourier single-shot turbo spin echo [23, 24], 3D gradient recalled echo (GRE) with breath-hold [25] and non-Cartesian acquisition sequences such as ultrashort echo time (UTE) imaging [26] and radial GRE [27]. In our work, we achieved a promising detection rate for all acceleration factors. Reaching 100% for the lowest acceleration CS-AI-7 and decreased to 96.9% for the fastest acceleration CS-AI-15. It should be emphasised that only one nodule in CS-AI-10 and two in CS-AI-15 with a size of 3 mm and 4 mm, respectively, were missed. This results in a detection rate of 100% for nodules larger than 6 mm. The detection rate in our work is therefore substantially higher than the 64% described by Yu et al and comparable to the radial GRE analysed in their work [27]. It should be emphasised that in our work the acquisition was performed in free breathing, as opposed to single breath hold, potentially reducing strong motion artefacts of breath-hold acquisition. For a potential application in lung cancer screening, the Lung-RADS categories were evaluated for all MR sequences and the CT. An almost perfect agreement between the MR sequences and the CT was found. Most notably, individual downgrades of 4X nodules were shown for all sequences, which indicates insufficient morphologic detail. According to Lung-RADS, solid or part-solid nodules under 6 mm are reliably classified as benign (Lung-RADS 2) and do not lead to a change in the screening process. This also applies to nodules of less than 4 mm that occur during the annual follow-up examination. Consequently, CS-AI-10 would be a suitable acceleration that achieves a sufficient detection rate with a scan time of 2:34 min, which corresponds to a reduction of 34% compared to CS-AI-7.

Despite the promising results, our work has several limitations. Although the cohort size in this study is respectable in regard to its prospective character and elaborated scanning protocol, the most important limitation is still the number of patients and low number of part-solid and ground glass nodules. However, the part-solid nodules in our cohort were detected in all sequences. Compared to a normal screening cohort, the pathological findings were overrepresented, which could have affected reader performance. In accordance with the Lung-RADS recommendation, our analysis was limited to the maximum diameter; volumetric measurements, which are considered more reproducible, will prospectively have an impact on the future evaluation of pulmonary nodules [28]; however, they are not included in our work and need to be analysed in the future studies. Previous studies have shown that the development of non-cartesian acquisition sequences like UTE leads to promising results. However, there are no comparative studies with sequences using CS and AI reconstruction, which need to be investigated.

Overall, the combination of compressed sense scanning and AI enables a significant reduction in scan time for pulmonary MRI while maintaining a high detection rate of pulmonary nodules. Besides the sole detection rate, nodule characteristics stay comparable to the gold standard CT, setting an intermediate acceleration factor ideally between clinically feasible scan times and high diagnostic quality. While our results suggest that MRI could be a valuable tool in lung cancer screening, further research and validation studies are needed to establish its practical application and long-term efficacy.

## Supplementary information

ELECTRONIC SUPPLEMENTARY MATERIAL

## Abbreviations

AI: Artificial intelligence
CS: Compressed sensing
CS-AI: Compressed sensing and artificial intelligence
CT: Computed tomography
GRE: Gradient recall echo
ICC: Intraclass correlation coefficient
MRI: Magnetic resonance imaging
UTE: Ultrashort echo time
