# Supplementary material for: Speed and efficiency: evaluating pulmonary nodule detection with AI-enhanced 3D gradient echo imaging
Source: Eur Radiol. 2024 Aug 18;35(4):2237–44. doi: 10.1007/s00330-024-11027-5 (PMC11914225; doi:10.1007/s00330-024-11027-5)
Supplement: Supplementary file 1 — ELECTRONIC SUPPLEMENTARY MATERIAL [file 330_2024_11027_MOESM1_ESM.pdf]

Speed and Efficiency: Evaluating Pulmonary Nodule Detection with AI-Enhanced 3D Gradient Echo Imaging
ELECTRONIC SUPPLEMENTARY MATERIAL

S1: Post-hoc analysis (pairwise wilcoxon test):
Main pulmonary arteries
CS-AI 7 vs. 10 $p<0.001$
CS-AI 7 vs. 15 $p<0.001$
CS-AI 10 vs. 15 $p=0.243$

Segmental vessels
CS-AI 7 vs. 10 $p<0.01$
CS-AI 7 vs. 15 $p<0.001$
CS-AI 10 vs. 15 $p=0.144$

Subsegmental vessels
CS-AI 7 vs. 10 $p<0.01$
CS-AI 7 vs. 15 $p<0.001$
CS-AI 10 vs. 15 $p=0.480$

Peripheral 1/3 pulmonary vessels
CS-AI 7 vs. 10 $p<0.01$
CS-AI 7 vs. 15 $p<0.001$
CS-AI 10 vs. 15 $p=0.121$

Pleura and subpleural vessels
CS-AI 7 vs. 10 $p<0.001$
CS-AI 7 vs. 15 $p<0.001$
CS-AI 10 vs. 15 $p=0.290$
